# Traditional herbal formula Wu-Mei-Wan alleviates TNBS-induced colitis in mice by inhibiting necroptosis through increasing RIPK3 *O*-GlcNAcylation

**DOI:** 10.1186/s13020-021-00493-4

**Published:** 2021-08-16

**Authors:** Fan Wu, Qingqing Shao, Zhe Cheng, Xinyu Xiong, Ke Fang, Yan Zhao, Ruolan Dong, Lijun Xu, Fuer Lu, Guang Chen

**Affiliations:** 1 Institute of Integrated Traditional Chinese and Western Medicine, Tongji Hospital, Tongji Medical College, Huazhong University of Science and Technology, Wuhan, 430030 China; 2 Department of Integrated Traditional Chinese and Western Medicine, Tongji Hospital, Tongji Medical College, Huazhong University of Science and Technology, Wuhan, 430030 China

**Keywords:** Wu-Mei-Wan, Colitis, Necroptosis, *O*-GlcNAcylation, RIPK3

## Abstract

**Background:**

Accumulating evidence indicated that necroptosis plays an essential role in the pathogenesis of inflammatory bowel disease (IBD). The *O*-linked β-*N*-acetylglucosaminylation (*O*-GlcNAcylation) of necroptotic signal molecule receptor-interacting serine-threonine kinase 3 (RIPK3) was reported to exert a protective effect in gut inflammation. Our recent study suggested traditional Chinese herbal formula Wu-Mei-Wan (WMW) as an effective prescription in mouse colitis. However, the potential mechanisms are not fully understood. Considering the crucial role of necroptosis in the pathogenesis of IBD, therefore, this study was designed to explain whether the anti-colitis effect of WMW is mediated by modulating necroptosis and its related mechanisms.

**Methods:**

The protective effects of WMW on colitis have been determined by detecting colitis mice body weight, disease activity index (DAI), survival rate and colon length. Colonic inflammation was examined by inflammatory cells infiltration and local cytokines levels. After then, we measured the levels of necroptosis and *O*-GlcNAcylation. C* O*-immunoprecipitation experiments were used to address whether elevated *O*-GlcNAcylation can inhibit necroptotic signal transduction in the treatment of WMW. Finally, the key enzymes in *O*-GlcNAcylation: *O*-GlcNAc transferase (OGT) and *O*-GlcNAcase (OGA) were examined and molecular docking analysis was used to determine effective natural compounds in the regulation on OGT and OGA activities.

**Results:**

Our results showed that WMW significantly improved mice body weight, survival rate and colon length, decreased DAI in TNBS-induced colitis. WMW obviously alleviated colonic inflammatory responses with reduced macrophages, neutrophils infiltration and local IL-1β, IL-6, TNF-α and IFN-γ levels. It was found that WMW increased colonic *O*-GlcNAcylation level and inhibited the activation of RIPK1, RIPK3 and MLKL. Then, further experiments revealed that WMW enhanced OGT activity and suppressed OGA activity, thereby increasing RIPK3 *O*-GlcNAcylation and inhibiting the binding of RIPK3 and MLKL, which led to the inhibition of necroptosis. Additionally, docking analysis demonstrated that hesperidin, coptisine and ginsenoside Rb1 may exert a major role in the regulation on OGT and OGA activities by WMW.

**Conclusion:**

Our work demonstrated that WMW can alleviate TNBS-induced colitis in mice by inhibiting necroptosis through increasing RIPK3 *O*-GlcNAcylation.

## Introduction

In the past decade, inflammatory bowel disease (IBD), which consist of Crohn's disease (CD) and ulcerative colitis (UC), has emerged as a public health challenge worldwide [1]. It was estimated that more than 1.5 million residents in the USA and 2.5 million in Europe suffer from IBD [2], and the incidence is now also growing rapidly in many newly industrialized countries in Asia, Africa, South America and the Middle East [3, 4]. IBD is a kind of chronic idiopathic gastrointestinal inflammatory disorder with unclear etiology. To date, studies have suggested that multiple factors may participate in its pathogenesis, such as genetic susceptibility, dysregulated gut microbiota, mucosal barrier impairment and environmental factors [5]. In clinic, current main treatments for IBD including aminosalicylates, corticosteroids, immunomodulators, anti-TNF-α drugs and surgery [6]. However, these approaches do not bring satisfactory effects due to its limited efficacy and related side effects, thus it is still difficult to maintain durable remission in IBD. Hence, it is urgent and important to further develop more effective treatments for IBD.

Recent research demonstrated that necroptosis, a newly recognized programmed necrosis, plays an important role in the pathogenesis of IBD [7]. Necroptosis is considered as a typically highly proinflammatory cell death mode that critically depends on receptor-interacting serine-threonine kinase 3 (RIPK3) and mixed lineage kinase domain-like (MLKL) [8]. Different from apoptosis, necroptosis is a caspase independent cell death but inhibited by caspase activity [9]. Under TNF-α stimulation, TNF receptor 1 (TNFR1) ligation recruits an early complex composed of TNFR1-associated death domain protein (TRADD) and RIPK1. Subsequently, RIPK1 is activated by deubiquitylation and phosphorylation [10]. In the absence of caspase 8 activity, phosphorylated RIPK1 recruits and activates RIPK3 via homotypic RIP homology interaction motif-domain (RHIM), and then MLKL is also recruited and phosphorylated by p-RIPK3, and eventually form a complex called the necrosome. Within this necrosome, phosphorylated MLKL forms active oligomers that translocate to the plasma membrane and destabilize it, which finally disrupting cellular membrane integrity [11, 12]. The cell then dies by necroptosis and generally manifests with morphological features of necrosis, which including cell swelling, membrane disruption and the release of the intracellular damage-associated molecular patterns (DAMPs) that promote inflammation [13]. Many clinical studies have verified that elevated necroptosis was strongly associated with IBD intestinal inflammation and contributed to strengthen the disease activity [14–16]. Conversely, the pharmacological or genetic inhibition of necroptosis attenuates the pathological changes and improves the disease conditions in colitis mice [17–19]. Therefore, inhibiting necroptosis has become a promising strategy against IBD.

Given RIPK3’s essential role in the signal transduction during necroptosis, inhibiting RIPK3 activity has been extensively explored by many researchers as a direction for suppressing necroptosis and proved effective [20]. In addition to phosphorylation, it was found that the activation of RIPK3 is also affected by multiple post-translational modifications (PTM), among which *O*-linked β-*N*-acetylglucosaminylation (*O*-GlcNAcylation) has gained more and more attention [21]. Intriguingly, *O*-GlcNAcylation occurs on the same Ser/Thr sites with phosphorylation, indicated that these PTMs can affect each other (mutually exclusive) [22]. The *O*-GlcNAcylation/phosphorylation of protein exhibit a competitive inhibition of its phosphorylation/*O*-GlcNAcylation. Whether this phenomenon exist in the process of RIPK3 activation? Various studies have proved that enhanced RIPK3 *O*-GlcNAcylation can effectively reducing p-RIPK3, inhibiting necroptosis [21, 23, 24]. Mechanically, the cycling of *O*-GlcNAc modifications is achieved by a single pair of enzymes: *O*-GlcNAc transferase (OGT) and *O*-GlcNAcase (OGA). OGT attaches *O*-GlcNAc to proteins while OGA specifically removes *O*-GlcNAc from proteins [25]. Studies demonstrated that the level of *O*-GlcNAc modification in intestinal epithelial cells (IECs) of IBD patients showed a significant reduction when compared to healthy subjects [26], and genetic deletion of OGT induced the colonic inflammation in mice due to the reduction of RIPK3 *O*-GlcNAcylation level [26, 27]. Taken together, increasing RIPK3 *O*-GlcNAcylation can reduce RIPK3 activation, thereby inhibiting necroptosis, finally alleviating colitis.

IBD belongs to the “Changpi” or “chronic dysentery” in the theory of traditional Chinese medicine (TCM). Wu-Mei-Wan (WMW) is a famous classic Chinese herbal formula, which includes ten herbs: *Mume Fructus* (*Prunus mume* (Siebold) Siebold & Zucc), *Asari Radix et Rhizoma* (*Asarum heterotropoides f. mandshuricum* (Maxim.) Kitag.), *Zingiberis Rhizoma* (Zingiber officinale Roscoe), *Coptidis Rhizoma* (*Coptis chinensis* Franch.), *Angelicae Sinensis Radix* (*Angelica sinensis* (Oliv.) Diels), *Typhonii Rhizoma* (*Typhonium giganteum* Engl.), *Zanthoxyli Pericarpium* (*Zanthoxylum bungeanum* Maxim), *Cinnamomi Ramulus* (*Cinnamomum cassia* (L.) J. Presl), *Ginseng Radix et Rhizoma* (*Panax ginseng* C. A. Mey), and *Phellodendri Chinensis Cortex* (*Phellodendron chinense* C. K. Schneid.). Since ancient times, WMW has been widely applied for the treatment of IBD in China, and studies have confirmed that WMW has significant clinical effects [28, 29]. Nevertheless, until now, there is still a lack of corresponding research on the underlying mechanisms of WMW’s anti-colitis effects. Our recent study has demonstrated that WMW can prevent chronic colitis-related intestinal fibrosis, in which WMW showed an obvious anti-inflammatory activity [30]. However, the anti-inflammatory mechanism of WMW has not been further investigated in our last project and the potential effects and mechanisms of WMW on colon necroptosis is also remain unknown. Considering the crucial role of necroptosis in the pathogenesis of IBD, therefore, this study was designed to explain whether the anti-colitis effect of WMW is mediated by modulating necroptosis.

## Materials and methods

### Reagents and chemicals

Caspase-3, Caspase-8, RIPK1, RIPK3 (rabbit antibody), MLKL, OGT and OGA antibodies were purchased from Proteintech (Wuhan, China). Phospho-RIPK1, phospho-RIPK3, phospho-MLKL and secondary antibodies were from Cell Signaling Technology (Beverly, MA, USA). MPO antibody was provided by ABclonal Technology (Wuhan, China). CD68 antibody was from Abcam (Cambridge, MA). *O*-GlcNAc antibody was obtained from Santa Cruz Biotechnology (Santa Cruz, CA). Another RIPK3 (mouse) antibody was purchased from Novus Biologicals (Littleton, CO, USA). 2,4,6-trinitrobenzene sulfonic acid (TNBS) was from Thermo Fisher Scientific (CA, USA). Protein A/G magnetic beads were purchased form Bimake (Shanghai, China). The TUNEL staining and immunohistochemistry kits were purchased from Wuhan Gugeshengwu Technology Co.,Ltd. (Wuhan, China). All other regular reagents were from Wuhan Gugeshengwu Technology Co.,Ltd. unless otherwise specified.

### WMW preparation and quality control

WMW decoction was prepared as we previously described [30]. In brief, except for *Typhonii Rhizoma,* which was boiled for 4 h, all other herbs were boiled together for 2 h. Then, combining these two herb extracts and concentrating it to 1.92 g/mL to acquire WMW decoction. The composition ratio and week usage of WMW was shown in Table 1. In addition, the quality control for WMW has also been determined by high-performance liquid chromatography (HPLC) fingerprinting analysis.

**Table 1:** The composition of WMW

Herbal medicine	Occupied percent	Week usage (g)
Mume Fructus	20%	19.2
Asari Radix et Rhizoma	7.5%	7.2
Zingiberis Rhizoma	12.5%	12
Coptidis Rhizoma	20%	19.2
Angelicae Sinensis Radix	5%	4.8
Typhonii Rhizoma	7.5%	7.2
Zanthoxyli Pericarpium	5%	4.8
Cinnamomi Ramulus	7.5%	7.2
Ginseng Radix et Rhizoma	7.5%	7.2
Phellodendri Chinensis Cortex	7.5%	7.2

### Animal experiment

**Fig. 1:**
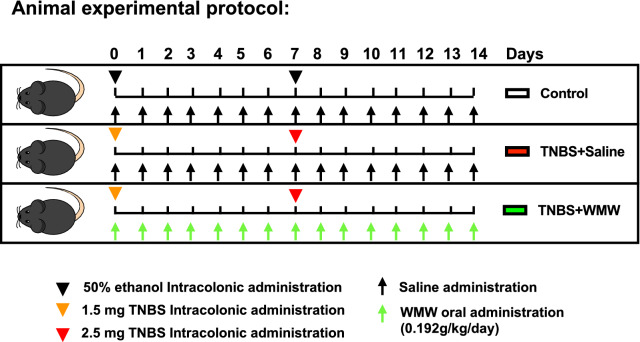
Animal experimental protocol. After 1-week acclimation, the mice were randomly divided into three groups: control group, model group and WMW group. Control group: The mice were subjected to 50% ethanol intracolonic administration and treatment with 0.9% saline. Model group: The mice were subjected to TNBS intracolonic administrations and treatment with 0.9% saline. WMW group: The mice were subjected to TNBS intracolonic administrations and treatment with WMW. All mice were gavaged daily started with the first intracolonic administration induction and continued until the end of the study

The animal experiment was reviewed and approved by the Animal Ethics Committee of Tongji Medical College, Huazhong University of Science and Technology (HUST) before and during the experiment. Male seven-week-old C57BL/6 mice were obtained from Hubei Province Experimental Animal Research Center (SPF-grade) and housed in the experimental animal center of Tongji medical college under conditions of 12 h dark/light cycle, 60 ± 5% relative humidity and 20 ± 2 °C environmental temperature. After one-week acclimation, the mice were randomly divided into three groups: control group (n = 12), model group (n = 12) and WMW group (n = 12). The mice were fasted overnight with free access to water before TNBS or 50% ethanol intracolonic administration, which was achieved by inserting a polyethylene catheter (2 mm in outer diameter) 3–4 cm into rectum under anesthesia. In detail, the mice of control group were injected intracolonically with 50% ethanol, while the mice of model and WMW groups were injected intracolonically with 100 μl of 50% ethanol containing a TNBS solution. For inducing acute colitis, TNBS was administered twice, the first week is 1.5 mg and the second week is 2.5 mg. In WMW group, WMW treatment started with the first TNBS induction and continued until the end of the study, while the mice of control and model groups was gavaged daily with 0.9% saline in the same dose. The detailed animal experimental protocol is illustrated in Fig. 1. At the end of the experiment, all mice were anaesthetized with 1% pentobarbital (65 μl/10 g, i.p.). After collecting blood sample, the mice were euthanized with CO_2_.

### Disease activity index (DAI) and histological scoring

During the study, the mice were monitored and weighed daily. For calculating disease activity index (DAI), we recorded body weight loss, stool consistency and rectal bleeding scores. For body weight loss, 0 points for no weight loss, weight losses in the ranges between 1 and 5%, 6 and 10%, 11 and 20%, and beyond thereafter from baseline were assigned as 1, 2, 3, and 4 points, respectively. For stool consistency and rectal bleeding, 0 was assigned for well-formed pellets/no blood, 2 points for pasty and semiformed stools/ positive bleeding, and 4 points for liquid stools/gross bleeding. The sum of the scores of above parameters was expressed as DAI.

Histological scores were examined according to previously studies [30, 31]. Briefly, 3 independent parameters—extent, inflammation, and crypt damage were used to evaluate the degree of histological damage. For extent, the depth of lesions between none (no lesion), mucosa, submucosa and transmural were assigned as 0, 1, 2, and 3 points. For inflammation, the severity of inflammation between none (no inflammation), slight, moderate and severe were assigned as 0, 1, 2, and 3 points. For crypt damage, the degree of damage between none (no damage), basal one-third lost, basal two-thirds lost, only surface epithelium intact and entire crypt and epithelium lost were assigned as 0, 1, 2, 3 and 4 points. The sum of the above parameter scores were multiplied by scores of spreads that in the ranges between 0–25%, 26–50%, 51–75% and 76–100% were assigned as 1, 2, 3, and 4 points, respectively. The final multiplied score was a histological score (0–40).

## Biochemical analysis

Serum aspartate aminotransferase (GOT) and alanine aminotransferase (GPT) levels were tested by using corresponding assay kits from Nanjing Jiancheng Bioengineering Institute (Nanjing, China). For detecting colonic inflammatory cytokines, fresh colon tissues were extracted using RIPA buffer and the supernatants were collected. The levels of colonic IL-1β, IL-6, TNF-α and IFN-γ were determined by ELISA kits (Bio-Swamp, Wuhan, China) according to the manufacturer’s instructions.

### Immunohistochemistry and immunofluorescence staining

Paraffin-embedded colon slides were dewaxed and rehydrated according to the standard protocol. After that, endogenous peroxidase activity was blocked with 3% H_2_O_2_ solution after antigen retrieval. Then, the slides were block with 10% normal goat serum for 1 h. Primary antibodies were applied overnight at 4 °C followed by the incubation of HRP-conjugated secondary antibody for 1 h at room temperature. The slides were visualized by DAB and counterstained with hematoxylin. Images were captured by an Olympus BX51 system and analyzed by Image J software (National Institutes of Health, USA).

For immunofluorescence staining, the slides followed the same steps as immunohistochemistry staining, except that they do not need to be incubated with H_2_O_2_ solution. Alexa Flour 488 or CY3-conjugated secondary antibody was applying after primary antibodies incubation. The nuclei were stained using DAPI solution and all operations were carried out under the light-protected conditions after incubating the secondary antibody. The images were taken by Olympus BX51 fluorescent microscope system.

### TUNEL (Terminal dexynucleotidyl transferase (TdT)-mediated dUTP nick end labeling) staining

Colon slides were dewaxed and rehydrated as above mentioned. Slides were digested with Proteinase K at 55 °C for 1 h and stained using a TUNEL apoptosis kit (Gugeshengwu, Wuhan, China) according to manufacturer’s instructions. An Olympus BX51 system was used to observe TUNEL-positive cells.

### Western blot analysis

Proteins from colon tissues were extracted by RIPA buffer supplemented with 1% of PMSF and protease inhibitor cocktail followed the standard protocol, and then protein concentrations were quantified using a bicinchoninic acid (BCA) protein assay kit. Protein samples (40 μg/lane) were loaded on SDS-PAGE gel and electrotransferred to a 0.45 μm nitrocellulose membrane (280 mA, 1 kDa/min). The membranes were blocked with 5% nonfat milk for 1 h and incubated with primary antibodies overnight at 4 °C. Then, fluorescence-conjugated secondary antibodies were applied to the membranes for 1 h at room temperature. The membranes were visualized with Odyssey Infrared Imaging (LI-COR Biosciences, USA). Target proteins were normalized to β-actin and quantified by Image J software.

### Co-immunoprecipitation

Co-immunoprecipitation (Co-IP) analysis was as described previously [32]. In brief, frozen colon tissues were homogenized in ice-cold RIPA buffer contained 1% PMSF and protease inhibitor cocktail and then the supernatants were collected. At the same time, protein A/G magnetic beads were prepared. IP antibody or its isotype IgG was added to the bead slurry to form bead-antibody complexes. After that, the supernatants were incubated with bead-antibody complexes overnight at 4 ◦C. After washing with the elution buffer, the protein complexes were boiled and subjected to western blot as described previously. Corresponding IgG control should be set for each sample. In our study, due to the close molecular weight between RIPK3 and IgG heavy chain, we used two different host RIPK3 antibodies in IP process and western blot process to avoid the influence of isotype IgG heavy chain in western blot process.

### High-performance liquid chromatography fingerprinting

The main chemical constituents of WMW extract was determined by HPLC fingerprinting. WMW extract was dissolved in water at a concentration of 1.92 g/ml (w/v) and further diluted with methanol–water (50:50) to 0.96 g/ml (w/v). Samples were passed through an Acclaim™ 120 C18 column (4.6 mm × 250 mm, 5 μm) at a flow rate of 1.0 ml/min with the mobile phases of methanol (A) and 0.1% phosphoric acid (B). HPLC signals were recorded at 240 nm detection wavelength with gradient eluting detailed in Table 2.

**Table 2:** Mobile phase condition of HPLC

Times (min)	Methanol (A)	0.1% Phosphoric acid
0	8	92
120	65	35
121	8	92
135	8	92

### Molecular docking analysis

The 3D structure of OGT (ID: 4GYY) and OGA (ID: 5UN9) was obtained from Protein Data Bank (PDB), and the structure of herb compounds was download from PubChem. Briefly, AutoDockTools 1.5.6 was used to process ligands and receptors, and AutoDock Vina 1.1.2 was applied for molecular docking and analysis of docking results. The docking energy value was determined by the consistency score function of the ligand-receptor affinity. For each ligand, the lowest binding energy and different conformations were recorded. PyMOL software was applied for the final visualization.

### Statistical analysis

All data are presented as means ± SEM. Statistical analyses were performed followed this rule: Firstly, the normality of data is tested by Shapiro–Wilk test; Secondly, data fits the normal distribution are tested for homogeneity of variance via one-way ANOVA; Finally, Tukey’s multiple comparisons test can be performed to compare multiple groups only if there is no significant variance inhomogeneity between groups. Mann–Whitney U test should be used as an alternative post hoc test when the data show non-normality or variance inhomogeneity. Statistics were analyzed using the GraphPad Prism 8 software, and *p* < 0.05 was considered as statistically significant.

## Results

### WMW significantly alleviates TNBS-induced colitis in mice

**Fig. 2:**
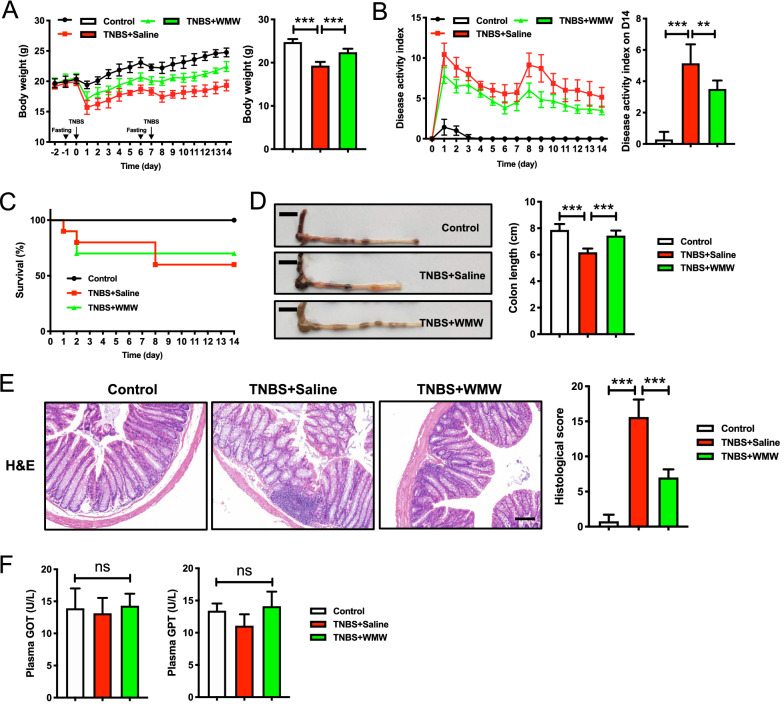
WMW significantly alleviates TNBS-induced colitis in mice. **A** Body weight of mice was recorded daily during the experiment. (n = 6–8). **B** Disease activity index (DAI) of mice was recorded daily during the experiment. (n = 6–8). **C** Survival curve of different groups during the experiment. **D** Representative colon images of different groups. Scale bar, 1 cm (n = 6–8). **E** Representative colon H&E staining and the histological score of different groups. Scale bar, 200 μm. (n = 6–8). **F** Serum GOT and GPT levels of different groups. (n = 6–8). All data are presented as means ± SEM. *p < 0.05, ** *p* < 0.01, ***p < 0.001

In our previous study, three different doses of WMW were used for colitis mice, in which high-dose WMW showed the best therapeutic effects [30]. So, based on that, we only applied single high-dose WMW in this work to better explore the potential mechanisms. To demonstrate the protective effects of WMW on TNBS-induced colitis, we measured mice body weight, DAI, survival rate and colon length. As expected, compared to model group, WMW treatment decreased colitis-induced weight loss in mice (Fig. 2A). It was observed that WMW significantly reduced DAI during experiment and increased survival rate at the end of experiment (Fig. 2B, C). Similarly, the mice of WMW group also showed longer colon length than that of model group (Fig. 2D). Meanwhile, colonic H&E examination revealed that colitis mice showed typical inflammatory cell infiltration and histological damage compared to control group, while WMW treatment exerted a significant protective effect in colitis (Fig. 2E). All of these data suggested that WMW can significantly alleviates TNBS-induced colitis in mice. Additionally, we also tested serum levels of GOT and GPT in three groups, which proved that the application of WMW was non-toxic in this animal models (Fig. 2F).

### WMW reduces colonic inflammatory responses in TNBS-induced colitis

**Fig. 3:**
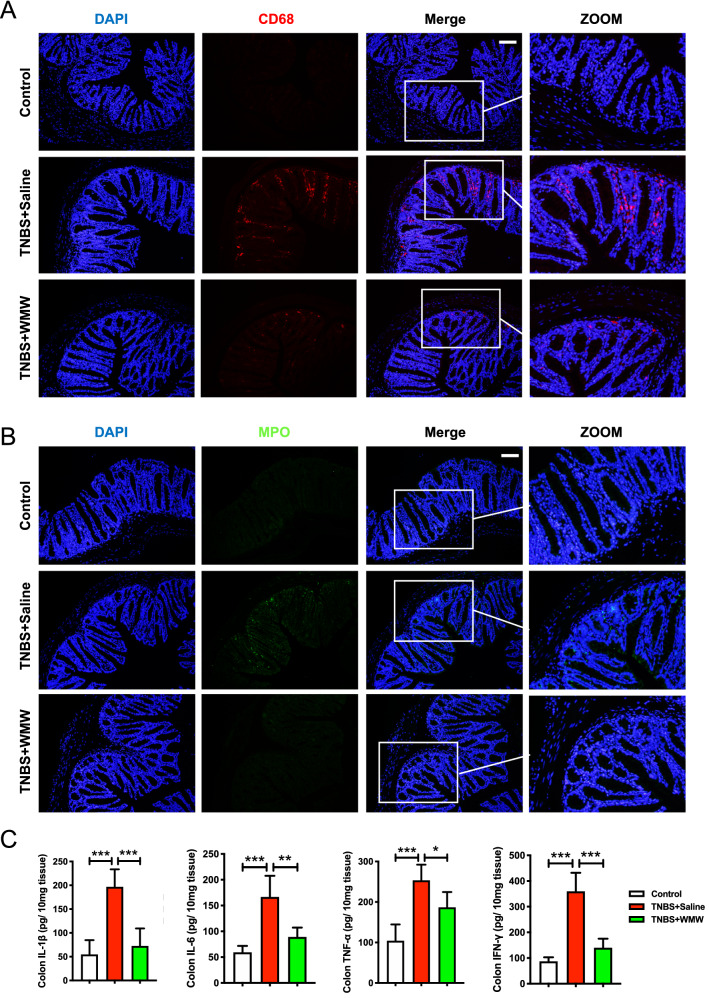
WMW reduces colonic inflammatory responses in TNBS-induced colitis. **A** Representative immunofluorescence staining for CD68 (macrophages) of different groups. Scale bar, 50 μm. (n = 4–6). **B** Representative immunofluorescence staining for MPO (neutrophils) of different groups. Scale bar, 50 μm. (n = 4–6). **C** Colonic inflammatory cytokines levels, including IL-1β, IL-6, TNF-α and IFN-γ. (n = 6–8). All data are presented as means ± SEM. *p < 0.05, ** *p* < 0.01, ***p < 0.001

To investigate the effects of WMW on colonic inflammatory responses, we detected the levels of inflammatory cells infiltration and local inflammatory cytokines expression. As shown in Fig. 3A, B, it was apparent that more macrophages (CD68-positive) and neutrophils (MPO-positive) were observed in model group, whereas few inflammatory cells were detected in control group. Compared with model group, WMW group showed a significant reduction in these inflammatory cells. Then, the levels of inflammatory cytokines, including IL-1β, IL-6, TNF-α and IFN-γ were also determined by ELISA. Consistent with above changes, in model group, the levels of IL-1β, IL-6, TNF-α and IFN-γ were obviously elevated compared to control group, and WMW treatment significantly decreased their expression levels (Fig. 3C). Altogether, our findings indicated that WMW treatment reduces colonic inflammatory responses in TNBS-induced colitis.

### WMW alleviates TNBS-induced colitis by inhibiting necroptosis

**Fig. 4:**
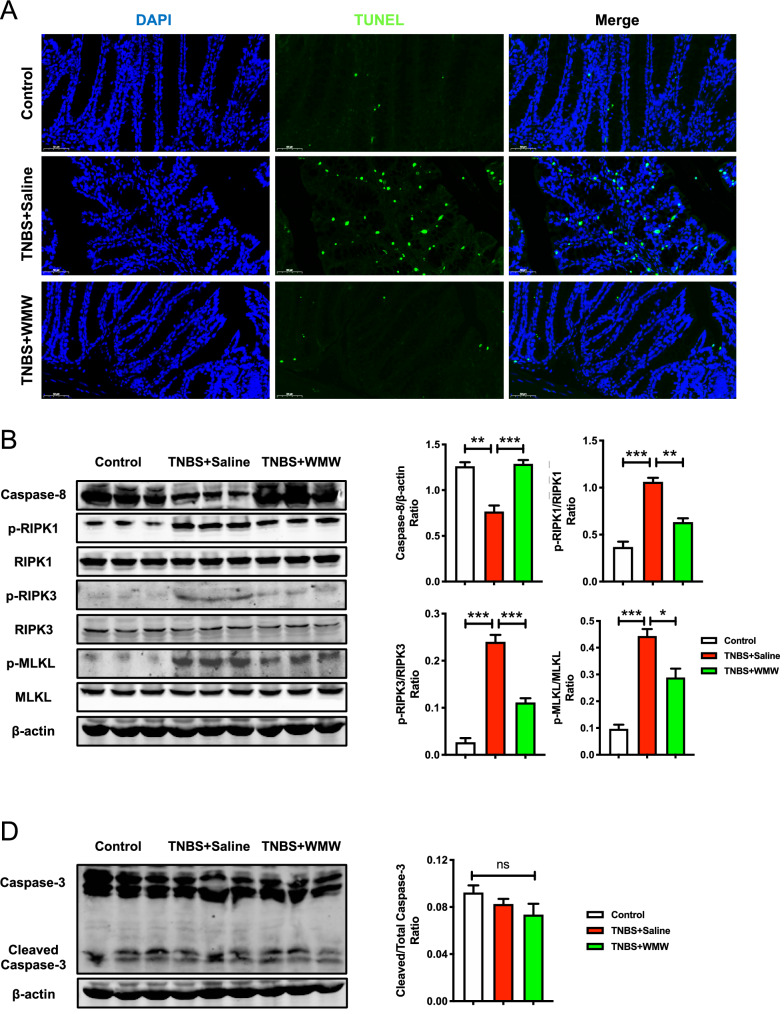
WMW alleviates TNBS-induced colitis by inhibiting necroptosis. **A** Representative TUNEL staining of different groups. Scale bar, 50 μm. (n = 4–6). **B** Representative western blots for caspase-8, p-RIPK1, RIPK1, p-RIPK3, RIPK3, p-MLKL and MLKL protein expressions in colon, and the quantification of caspase-8, p-RIPK1, p-RIPK3 and p-MLKL western blots. (n = 6). **C** Representative western blots for caspase-3 expression in colon, and the quantification of cleaved caspase-3 western blots. (n = 6). All data are presented as means ± SEM. *p < 0.05, ** *p* < 0.01, ***p < 0.001

To clarify our hypothesis whether the protective effect of WMW on colitis was related to the regulation of necroptosis. First, we tested the colonic level of cell death by TUNEL staining. Compared with control group, massive death of colonic cells was observed in model group, but compared with model group, treatment with WMW significantly decreased TUNEL-positive cells (Fig. 4A). This result preliminarily suggested that WMW can improve colitis-induced cell death. Due to TUNEL staining is not able to distinguish necroptosis from apoptosis, thus we further tested specific necroptotic and apoptotic markers though immunoblotting. As illustrated in Fig. 4B, compared with control group, caspase-8 activity was obviously decreased and the levels of p-RIPK1, p-RIPK3 and p-MLKL were remarkably increased in model group. It was found that WMW treatment significantly restored caspase-8 activity, inhibited the levels of p-RIPK1, p-RIPK3 and p-MLKL. These data indicated that WMW can effectively inhibit necroptosis in colitis. Interestingly, we also detected the level of cleaved caspase-3 (apoptotic markers) and found that there is no significant difference between the three groups (Fig. 4C). Collectively, all of these results demonstrated that necroptosis, but not apoptosis, contributed to TNBS-induced colitis, and WMW can alleviate TNBS-induced colitis by inhibiting necroptosis.

### WMW increases colonic *O*-GlcNAcylation level in TNBS-induced colitis

**Fig. 5:**
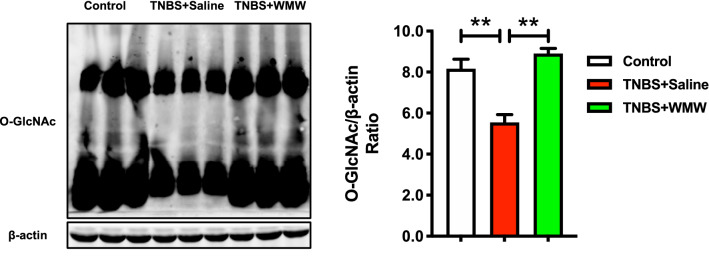
WMW increases colonic *O*-GlcNAcylation level in TNBS-induced colitis. Representative western blots for *O*-GlcNAc expression in colon, and the quantification of *O*-GlcNAc western blots. (n = 6). All data are presented as means ± SEM. *p < 0.05, ** *p* < 0.01, ***p < 0.001

Although our results have identified that WMW can inhibit necroptosis in TNBS-induced colitis. However, the potential mechanisms remain unknown. As mentioned before, *O*-GlcNAc modification can reduce the activation of necroptotic signal proteins (like RIPK3), thereby inhibiting necroptosis. Therefore, we measured the expression of *O*-GlcNAc to verify our idea. Compared with control group, the expression of *O*-GlcNAc was significantly decreased, which was consistent with previous studies [26, 27]. WMW treatment reversed above changes (Fig. 5). This revealed that WMW can increase colonic *O*-GlcNAcylation level, which may be associated with the inhibition of necroptosis.

### WMW promotes RIPK3 *O*-GlcNAcylation and inhibits the binding of RIPK3 and MLKL

**Fig. 6:**
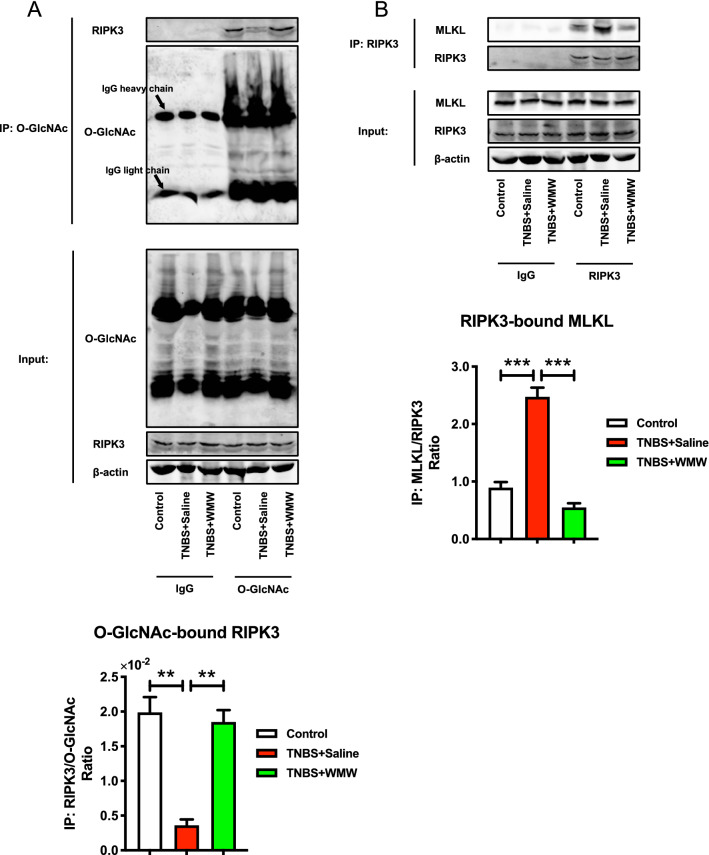
WMW promotes RIPK3 *O*-GlcNAcylation and inhibits the binding of RIPK3 and MLKL. **A** Representative western blots for immunoprecipitation of *O*-GlcNAc and RIPK3, and the binding level of *O*-GlcNAc and RIPK3. (n = 6). **B** Representative western blots for immunoprecipitation of RIPK3 and MLKL, and the binding level of RIPK3 and MLKL. (n = 6). All data are presented as means ± SEM. *p < 0.05, ** *p* < 0.01, ***p < 0.001

In order to further address the relationship between increased colonic *O*-GlcNAcylation and the inhibition of necroptosis, we applied co-immunoprecipitation assay to determine whether elevated *O*-GlcNAc directly affected necroptotic signal transduction. First, we used anti-*O*-GlcNAc antibody immunoprecipitations, as shown in Fig. 6A, it was observed that *O*-GlcNAcylated RIPK3 is obviously decreased in model group and WMW treatment largely promoted the binding of *O*-GlcNAc and RIPK3. This result verified that WMW increases RIPK3 *O*-GlcNAcylation, thereby inhibiting RIPK3 phosphorylation level. Based on that, then we further investigated the downstream signal transduction of necroptosis. The results of anti-RIPK3 antibody immunoprecipitations indicated that the RIPK3-MLKL complex formation in model group was higher than that of control group, and WMW treatment significantly inhibited the binding of RIPK3 and MLKL, reduced necrosome formation (Fig. 6B). These results demonstrated that WMW promotes RIPK3 *O*-GlcNAcylation, thereby reducing RIPK3 activation and inhibiting the necrosome formation, eventually lead to inhibition of necroptosis.

### WMW regulates OGT and OGA activities in TNBS-induced colitis

**Fig. 7:**
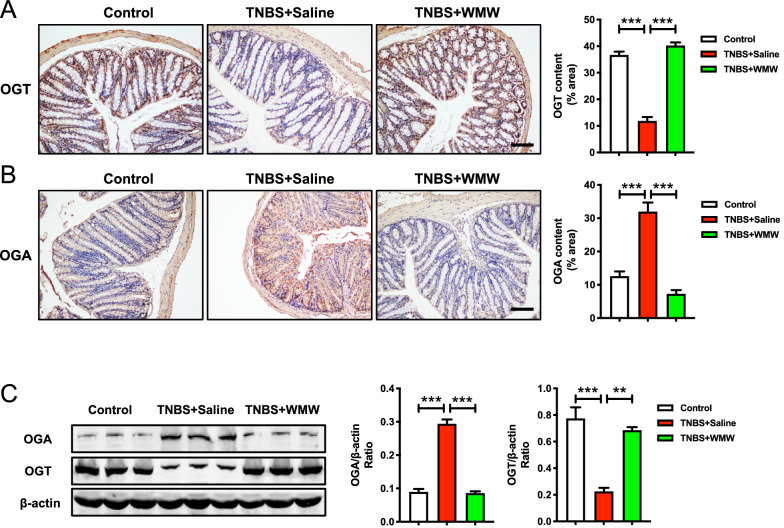
WMW regulates OGT and OGA activities in TNBS-induced colitis. **A** Representative immunohistochemistry staining for OGT of different groups, and the quantification of OGT immunohistochemistry staining. Scale bar, 100 μm. (n = 4–6). **B** Representative Immunohistochemistry staining for OGA of different groups, and the quantification of OGA immunohistochemistry staining. Scale bar, 100 μm. (n = 4–6). **C** Representative western blots for OGT and OGA protein expressions in colon, and the quantification of OGT and OGA western blots. (n = 6). All data are presented as means ± SEM. *p < 0.05, ** *p* < 0.01, ***p < 0.001

From above results, we can conclude that WMW alleviates TNBS-induced colitis by inhibiting necroptosis through increasing RIPK3 *O*-GlcNAcylation. How does WMW promote RIPK3 *O*-GlcNAcylation? This question gained our interest. As introduced before, OGT and OGA together control the dynamic cycling of *O*-GlcNAc modification. Hence, we determined the expression levels of OGT and OGA to explore the underlying mechanisms. Immunohistochemistry staining indicated that OGT level was significantly reduced while OGA was obviously elevated in model group, which confirmed that colitis leads to a reduction in colonic *O*-GlcNAcylation level (Fig. 7A, B). Compared with model group, WMW treatment significantly increased OGT level and suppressed OGA activity, which recovered colonic *O*-GlcNAcylation level (Fig. 7A, B). These results were also supported by immunoblotting (Fig. 7C). Taken together, these data demonstrated that the promotive effect of WMW on RIPK3 *O*-GlcNAcylation may be mediated by its regulation on OGT and OGA activities.

### Molecular docking reveals the potential compounds that regulate OGT and OGA activities

**Fig. 8:**
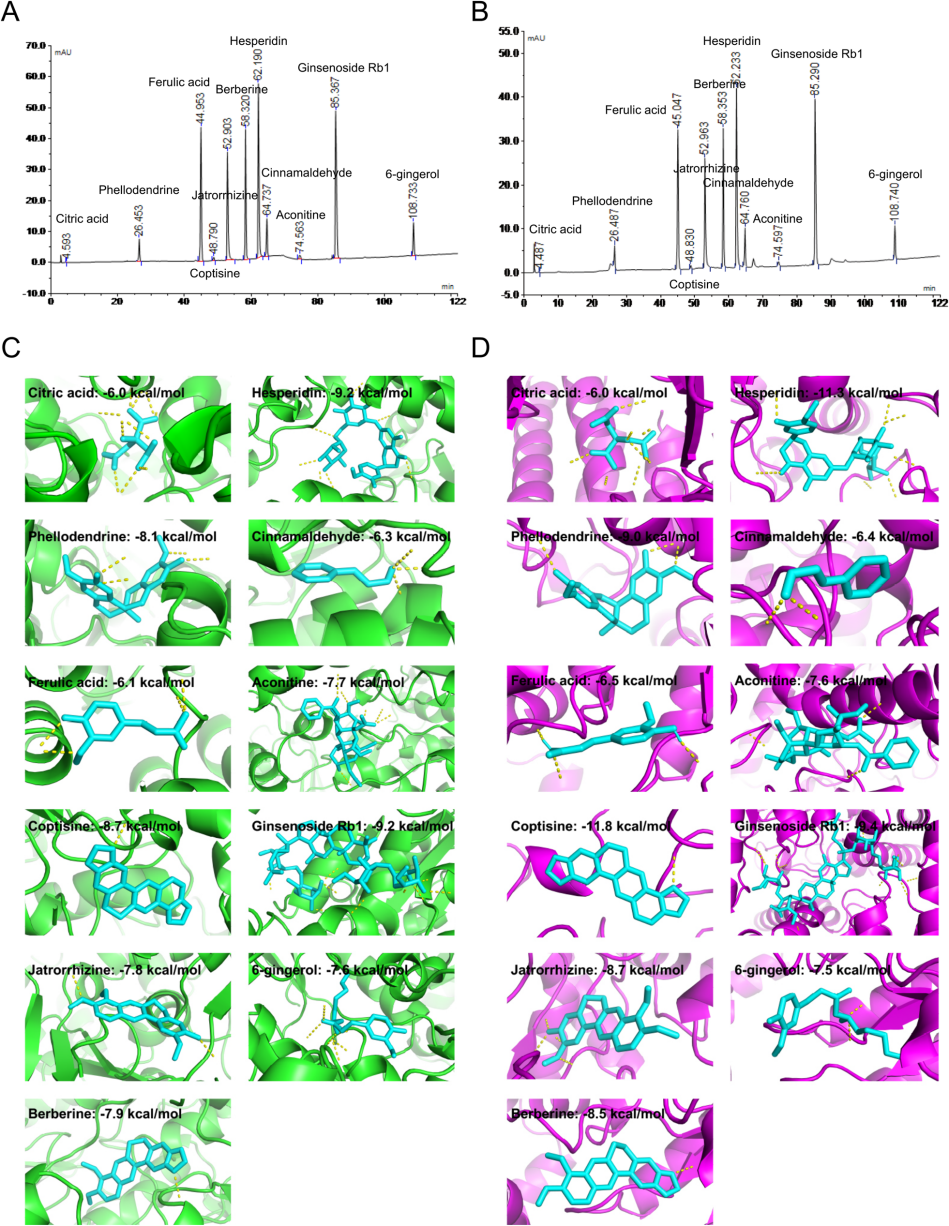
Molecular docking reveals the potential compounds that regulate OGT and OGA activities. **A** HPLC fingerprint chromatograms of the reference standards. **B** HPLC fingerprint chromatograms of the WMW extracts. **C** The interaction of WMW compounds and OGT by molecular docking. Yellow dotted line represents the hydrogen bond. **D** The interaction of WMW compounds and OGA by molecular docking. Yellow dotted line represents the hydrogen bond

To explore which compound in WMW possess the most important functions, we further examined the interactive activities of WMW compounds with the key enzymes OGT and OGA by molecular docking analysis. First, we have re-made HPLC analysis. A total of 11 maker compounds in WMW were determined by HPLC according to the identification standards of medicinal plants in Chinese Pharmacopoeia (2020 edition), including citric acid, phellodendrine, ferulic acid, coptisine, jatrorrhizine, berberine, hesperidin, cinnamaldehyde, aconitine, ginsenoside Rb1 and 6-gingerol (Fig. 8A, B). Then, the binding potency of these compounds with OGT and OGA was tested (Table 3). Among all, the top 3 compounds with OGT binding ability are hesperidin (− 9.2 kcal/mol), ginsenoside Rb1 (− 9.2 kcal/mol) and coptisine (− 8.7 kcal/mol), and the top 3 compounds with OGA binding ability are coptisine (− 11.8 kcal/mol), hesperidin (− 11.3 kcal/mol) and ginsenoside Rb1 (− 9.4 kcal/mol) (Fig. 8C, D). Based on these results, these data indicated that hesperidin, coptisine and ginsenoside Rb1 may exert a major role in the regulation on OGT and OGA activities by WMW.

**Table 3:** The estimated binding energy of active compounds

	**OGT**	**OGA**
Citric acid	− 6.0	− 6.0
Phellodendrine	− 8.1	− 9.0
Ferulic acid	− 6.1	− 6.5
Coptisine	− 8.7	− 11.8
Jatrorrhizine	− 7.8	− 8.7
Berberine	− 7.9	− 8.5
Hesperidin	− 9.2	− 11.3
Cinnamaldehyde	− 6.3	− 6.4
Aconitine	− 7.7	− 7.6
Ginsenoside Rb1	− 9.2	− 9.4
6-gingerol	− 7.6	− 7.5

## Discussion

**Fig. 9:**
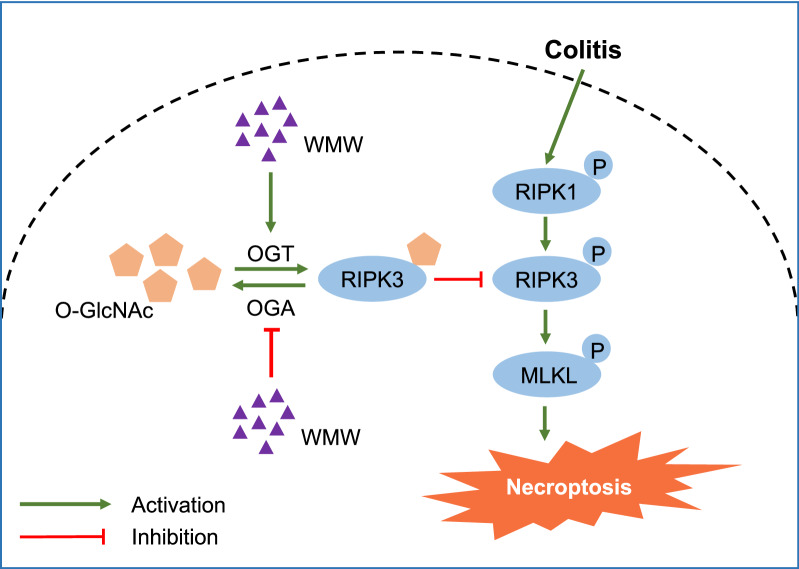
The potential role of WMW in TNBS-induced colitis in mice. WMW treatment can regulate colonic OGT and OGA activities, that is, enhance OGT activity and suppress OGA activity. These changes promote RIPK3 *O*-GlcNAcylation, thereby reducing RIPK3 activation and necrosome formation, and eventually inhibiting necroptosis and alleviating colitis

In this study, we demonstrated that the classic Chinese herbal formula WMW can alleviates TNBS-induced mice colitis, and the mechanisms involved may be as followed: WMW treatment can regulate colonic OGT and OGA activities, that is, enhance OGT activity and suppress OGA activity. These changes promote RIPK3 *O*-GlcNAcylation, thereby reducing RIPK3 activation and necrosome formation, and eventually inhibiting necroptosis and alleviating colitis (Fig. 9). Our work introduced an unexpected role of WMW in the inhibition of colitis-induced necroptosis.

Generally, cell death can be divided into two forms: programmed cell death (PCD) and uncontrolled cell death (UCD). Apoptosis was regarded as the typical form of PCD, whereas necrosis was seen as a classic UCD [33]. Currently, accumulating evidences have indicated that necrosis may be also a molecularly controlled regulated cell death, rather than a simple accidental cell death form [34]. Regulated necrosis consists of several cell death forms, like necroptosis, ferroptosis, parthanatos, pyroptosis, pyronecrosis and others [35], among which necroptosis and pyroptosis are the best characterized forms of regulated necrosis. Different from pyroptosis, which is induced by inflammasome activation, necroptosis is mediated by RIPK3 and MLKL. Various studies have revealed that necroptosis may be implicated in the pathogenesis of many human inflammatory diseases, particularly IBD, and the inhibition of necroptosis brings significant improvement in disease severity [36–39]. This point has been verified in our study.

Although the relationship between necroptosis and inflammation is not fully elucidated, necroptosis is still well-recognized as a potent inducer of inflammation due to the release of DAMPs in the process of necroptosis. Under sterile injury condition, endogenous proinflammatory molecules (DAMPs) are released by necrotic cells, then it can promote the activation of macrophages, dendritic cells and other sentinel cells, thereby increasing the level of abundant inflammatory cytokines including IL-1α, IL-1β, IL-18, IL-33, IL-36α, IL-36β, and IL-36γ [40, 41]. Additionally, due to the dysregulated gut microbiota in IBD [42], DAMPs can also activate proinflammatory reaction by combining with pathogen-associated molecular patterns (PAMPs) [43]. Recently, several studies suggested that RIPK1 and RIPK3 may have a proinflammatory function unrelated to necroptosis, like promoting NLRP3 inflammasome activation and IL-1β secretion, enhancing TNF secretion [44–47]. Given the precise mechanisms involved remain to be clarified, most scholars believed that the proinflammatory function of RIPK1 and RIPK3 is attributed to its activation in necroptosis. Anyway, in our study, WMW obviously decreased inflammatory cells infiltration and inflammatory cytokines expression accompanied with the inhibition of necroptosis. Therefore, it was justified to conclude that WMW alleviates colitis by inhibiting necroptosis. Of course, a genetic necroptosis-inhibition mouse (like RIPK3 knockout) model can further confirm this conclusion.

*O*-GlcNAcylation is a common protein PTM, it was reported that over 1000 proteins have been identified as target of this modification, including various signaling molecules and transcription factors [48]. To date, the role of *O*-GlcNAcylation on inflammation remains contradictory. In some conditions, *O*-GlcNAcylated proteins play the proinflammatory role, such as in diabetes, whereas other studies have documented that *O*-GlcNAcylation could exert the protective effects in some inflammatory diseases [49]. The potential explanation of this phenomenon may be as followed: The modification of different functional proteins in different conditions and different cell types may result in this phenomenon, which ultimately promote or inhibit inflammation. In IBD patients, the levels of *O*-GlcNAcylation and the expression of OGT, the enzyme promoting *O*-GlcNAcylation, were all reduced [26]. Meanwhile, in vivo studies have suggested the promotion of *O*-GlcNAcylation (such as OGT-transgenic mice) as an effective treatment in mice colitis [24, 27]. In contrast to these findings, some studies pointed that the colonic level of *O*-GlcNAc was increased in CD patients, and suppressing *O*-GlcNAcylation may protect mice from colitis [50, 51]. Collectively, the role of *O*-GlcNAcylation in gut inflammation of IBD is also not fully clear. More high-quality investigations are needed in this field. Here, in our work, we did observe a decrease in *O*-GlcNAcylation level in colitis mice, and WMW treatment significantly improved *O*-GlcNAcylation level, thereby alleviating mice colitis. How does elevated *O*-GlcNAcylation level alleviates gut inflammation in colitis? Based on limited research, the inhibition of necroptosis by hyper-*O*-GlcNAcylation-mediated RIPK3 inactivation may be the potential cause [21, 24]. Accordingly, we investigated the interaction between hyper-*O*-GlcNAcylation and necroptotic signal transduction, and the results verified our thoughts. After WMW treatment, RIPK3 *O*-GlcNAcylation level increased, which then led to a reduction in RIPK3 phosphorylation (RIPK3 inactivation), thereby inhibiting necroptosis and relieving colonic inflammation. After confirming the protective effects of WMW on colitis are mediated by its promotion of RIPK3 *O*-GlcNAcylation, we preliminarily explored the underlying mechanisms of WMW on RIPK3 *O*-GlcNAcylation. Since *O*-GlcNAcylation is regulated by OGT and OGA, we next detected the influence of WMW on these enzymes. Our results further supported above speculation: WMW treatment significantly enhanced OGT activity and suppressed OGA activity.

Although our data proved WMW as a potential regulator in OGT and OGA activities, but the research of its active compounds on this field are very lacking. To investigate which compound in WMW may play a major role in the regulation on OGT and OGA activities, we further conducted computational molecular docking analysis based on HPLC results. Hesperidin, coptisine and ginsenoside Rb1 showed the most potent binding potency both with OGT and OGA. Therefore, according to current evidence, hesperidin, coptisine and ginsenoside Rb1 are most likely to mediate the regulatory effects of WMW on OGT and OGA activities. Consistent with our results, it was reported that a natural derivative of hesperidin exerts a protective effect on DSS-induced mouse colitis by blocking epithelial cells necroptosis [18]. However, the research about coptisine and ginsenoside Rb1 on necroptosis or *O*-GlcNAcylation are lacking. Currently, few studies have explored the effects of active compounds of WMW on *O*-GlcNAc or *O*-GlcNAcylation. Therefore, there is a lot of space for the further investigations. This result laid the foundation for the later pharmacological studies on these natural compounds, and promoted the discovery of herb-derived anti-necroptosis natural products.

There are still some limitations of this work. First, in our study, we mainly focus on RIPK3 *O*-GlcNAcylation modification, and did not pay attention to other common glycosylation modifications. The reasons are followed: (i) Most proteins contain only one type of glycosylation [52]. (ii) *O*-GlcNAcylation occurs on the same Ser/Thr sites with phosphorylation, thereby exerting an inhibitory effect on proteins phosphorylation, while other glycosylation modifications occur on Asn side chains (through amide linkages) or C2 position of Trp (through C–C linkages) [53]. (iii) It is because recent authoritative studies have shown that RIPK3 *O*-GlcNAcylation effectively inhibits inflammation [21, 23], which inspired our ideas in this work. Meanwhile, there are no reports of other types of RIPK3 glycosylation so far [54]. Nevertheless, more precise research is more in line with scientific requirements. To fully uncover other types of glycoconjugates, mass spectrometry and nuclear magnetic resonance spectroscopy methods are needed. Subject to limited conditions, we did not adopt the above approaches in this study. Second, we did not experimentally verify the potential effects of hesperidin, coptisine and ginsenoside Rb1 on OGT and OGA activities in the colitis model. As we all know, it is hard to tell which compound exerts primary effects in herb formula due to its feature of multi-ingredients. To date, this is still an essential problem restricting the application and promotion of herb formulas. In the absence of a well-recognized high-throughput screening approach, we applied the widely used molecular docking technology to initially reveal the active compounds of WMW. In this study, in silico docking analysis suggested that hesperidin, coptisine and ginsenoside Rb1 are the most likely candidate compounds for regulating OGT and OGA activities based on current evidence. Although our findings provided significant clues on the potential of them, a same in vivo experimental verification is more convincing and perfect.

## Conclusions

In summary, this study explored the potential effects and underlying mechanisms of Wu-Mei-Wan on TNBS-induced colitis in mice. Our results demonstrated that WMW can enhance OGT activity and suppress OGA activity, thereby increasing RIPK3 *O*-GlcNAcylation, and then inhibiting necroptosis, eventually alleviating TNBS-induced colitis. According to current evidence, hesperidin, coptisine and ginsenoside Rb1 are the most likely candidate compounds for regulating OGT and OGA activities in this process.

## Data Availability

The data used and/or investigated during the present study are available from the corresponding author upon reasonable request.

## Abbreviations

WMW: Wu-Mei-Wan
IBD: Inflammatory bowel disease
CD: Crohn’s disease
UC: Ulcerative colitis
RIPK: Receptor-interacting serine-threonine kinase
MLKL: Mixed lineage kinase domain-like
TRADD: TNFR1-associated death domain protein
RHIM: RIP homology interaction motif-domain
DAMP: Damage-associated molecular pattern
PTM: Post-translational modifications
*O*-GlcNAc: *O*-Linked β-*N*-acetylglucosamine
*O*-GlcNAcylation: *O*-Linked β-*N*-acetylglucosaminylation
OGT: *O*-GlcNAc transferase
OGA: *O*-GlcNAcase
TCM: Traditional Chinese medicine
DAI: Disease activity index
PCD: Programmed cell death
UCD: Uncontrolled cell death
PAMP: Pathogen-associated molecular pattern
